# Modeling Not-Reached Items in Cognitive Diagnostic Assessments

**DOI:** 10.3389/fpsyg.2022.889673

**Published:** 2022-06-13

**Authors:** Lidan Liang, Jing Lu, Jiwei Zhang, Ningzhong Shi

**Affiliations:** ^1^Key Laboratory of Applied Statistics of MOE, School of Mathematics and Statistics, Northeast Normal University, Changchun, China; ^2^School of Mathematics and Statistics, Yili Normal University, Yining, China; ^3^Institute of Applied Mathematics, Yili Normal University, Yining, China; ^4^Faculty of Education, Northeast Normal University, Changchun, China

**Keywords:** cognitive diagnosis assessments, missing data mechanism, not-reached items, Bayesian analysis, sequential model

## Abstract

In cognitive diagnostic assessments with time limits, not-reached items (i.e., continuous nonresponses at the end of tests) frequently occur because examinees drop out of the test due to insufficient time. Oftentimes, the not-reached items are related to examinees’ specific cognitive attributes or knowledge structures. Thus, the underlying missing data mechanism of not-reached items is non-ignorable. In this study, a missing data model for not-reached items in cognitive diagnosis assessments was proposed. A sequential model with linear restrictions on item parameters for missing indicators was adopted; meanwhile, the deterministic inputs, noisy “and” gate model was used to model the responses. The higher-order structure was used to capture the correlation between higher-order ability parameters and dropping-out propensity parameters. A Bayesian Markov chain Monte Carlo method was used to estimate the model parameters. The simulation results showed that the proposed model improved diagnostic feedback results and produced accurate item parameters when the missing data mechanism was non-ignorable. The applicability of our model was demonstrated using a dataset from the Program for International Student Assessment 2018 computer-based mathematics cognitive test.

## Introduction

In educational and psychological assessments, examinees often do not reach the end of the test which may be due to test fatigue or insufficient time. The percentage of not-reached items in large-scale cognitive testing varies across individuals, items, and countries. According to the 2006 Program for International Student Assessment (PISA) study, an average of 4% of items are not reached (OECD, 2009). In the PISA 2015 (OECD, 2018) computer-based mathematics cognitive dataset, the percentage of not-reached items in Chinese Taipei is approximately 3%, and the percentage of not-reached items for the science cluster in a Canadian sample is 2% (Pohl et al., 2019). According to the PISA 2018 (OECD, 2021) computer-based mathematics cognitive data, the proportion of nonresponses for each item ranges from 0 to 17.3% in some countries, and the maximum percentage of not-reached items is as high as 5%. Thus, the missing proportion at the item level is relatively high. In addition, the percentage of nonresponses per nation (OECD countries) ranges from 4% to15% according to the PISA 2006 study (OECD, 2009). Even though the overall proportion of item nonresponses is small, the rate of not-reached responses for a single item or specific examinee may be large.

Previous literature focused on missing data in the item response theory (IRT) framework, which has shown that simply ignoring nonresponses or treating them as incorrect leads to biased estimates of item and person parameters (Lord, 1974, 1983; Ludlow and O’Leary, 1999; Huisman, 2000). Often, Rubin (1976) missing data mechanisms are worth reviewing for statistical inference. The complete data include observed data and unobservable missing data, and there are three types of missing data mechanisms (Rubin, 1976; Little and Rubin, 2002): missing completely at random (MCAR), missing at random (MAR), and not missing at random (NMAR). MCAR refers to the probability of missing data as independent of both observed and missing data. MAR refers to the probability of missing data as only dependent on observed data. NMAR refers to the probability of missing data as dependent on the unobserved missing data itself, which is not ignorable. In general, MCAR and MAR mechanisms do not affect the parameter estimations of interest or the followed-up inference, thus missing data can be ignored in these two specific missing data mechanisms. However, Rose et al. (2010, 2017) showed that the proportion of examinees’ correct scores based on the observed item responses was negatively correlated with the item nonresponse rate, which suggests that simple questions are easy to answer, and numerous difficult items may be omitted. Item nonresponses may depend on the examinee’s ability and the difficulty of the items, and therefore the ignorable missing data mechanism assumption (MCAR or MAR) becomes highly questionable. This leads to the development of measurement models that consider the NMAR mechanism. Specifically, several scholars have proposed multidimensional IRT (MIRT) models to handle missing responses (e.g., Holman and Glas, 2005; Glas and Pimentel, 2008; Pohl et al., 2019; Lu and Wang, 2020). For example, Glas and Pimentel (2008) used a combination of two IRT models to model not-reached items for speeded tests according to the framework of the IRT. Subsequently, Rose et al. (2010) proposed latent regression models and multiple-group IRT models for non-ignorable missing data. Debeer et al. (2017) developed two item response tree models to handle not-reached items in various application scenarios.

Recently, cognitive diagnosis (von Davier, 2008, 2018, 2014; Xu and Zhang, 2016; Zhan et al., 2018; Zhang et al., 2020) has received considerable attention from researchers because cognitive diagnostic test enables the evaluation of the mastery of skills or attributes of respondents and allows diagnostic feedback for teachers or clinicians, which in turn aids in decision-making regarding remedial guidance or targeted interventions. In addition, the cognitive diagnostic test has improved on traditional tests. General educational examinations only provide test or ability scores in large-scale testing. However, we can neither conclude that examinees mastered the knowledge nor understand why examinees answered questions incorrectly from a single score. Moreover, it is impossible to infer differences in knowledge state and cognitive structures between individuals with the same score. Thus, the information provided by traditional IRT is not suitable for the needs of individual learning and development. To date, numerous cognitive diagnostic models (CDMs) have been developed, such as the deterministic inputs, noisy “and” gate (DINA) model (de la Torre and Douglas, 2004; de la Torre, 2009); the noisy inputs, deterministic, “and” gate model (NIDA; Maris, 1999); the deterministic inputs, noisy “or” gate (DINO) model (Templin and Henson, 2006); the log-linear CDM (Henson et al., 2009); and the generalized DINA model (de la Torre, 2011). Subsequently, a higher-order DINA (HO-DINA) model (de la Torre and Douglas, 2004) was proposed to link latent attributes *via* higher-order ability. Furthermore, Ma (2021) proposed a higher-order CDM with polytomous attributes for dichotomous response data.

Numerous studies have focused on item nonresponses in IRT models (Finch, 2008; Glas and Pimentel, 2008; Debeer et al., 2017). However, only a few studies have discussed missing data in cognitive assessments. Ömür Sünbül (2018) limited missing data mechanisms to MCAR and MAR in the DINA model and investigated different imputation approaches for dealing with item nonresponses, such as coding item responses as incorrect and using person mean imputation, two-way imputation, and expectation-maximization algorithm imputation. Heller et al. (2015) argued that CDMs may have underlying relationships with knowledge space theory (KST), which has been explored in several previous studies (e.g., Doignon and Falmagne, 1999; Falmagne and Doignon, 2011). Furthermore, de Chiusole et al. (2015) and Anselmi et al. (2016) have developed models for KST to consider different missing data mechanisms (i.e., MCAR, MAR, and NMAR). However, in their work, missing response data may not have been handled effectively, which may have biased results. Shan and Wang (2020) introduced latent missing propensities for examinees in the DINA model. They also included a potential category parameter, which affects the tendency to miss items. However, they did not provide a detailed explanation of the category parameters. Moreover, their model did not distinguish the type of item nonresponses.

The confound of different types of missing data produces inaccurate attribute profile estimations, which consequently results in incorrect diagnostic classifications. To the best of our knowledge, there has been no model developed to date that describes not-reached items in cognitive diagnosis. Thus, a missing model for not-reached items is proposed to fill this gap in cognitive diagnosis assessments. Specifically, a higher-order DINA model is used to model responses and an IRT model to describe missing indicators, which is a sequential model with linear restrictions on item parameters (Glas and Pimentel, 2008). The model is connected by bivariate normal distributions between examinees’ latent ability parameters and missing propensity parameters and between item intercept and interaction parameters.

The rest of this paper is organized as follows. First, an IRT model is introduced as a missing indicator model for not-reached items. Then, a higher-order DINA model is used for the observed responses and the correlation between person parameters. Second, the Markov chain Monte Carlo (MCMC) algorithm (Patz and Junker, 1999; Chen et al., 2000) is developed to estimate the model parameters of the proposed model. Simulation studies are conducted to assess the performance of the proposed model for different simulation conditions. Third, a real dataset from the PISA 2018 (OECD, 2021) computer-based mathematics data is analyzed. Concluding remarks and future perspectives are provided thereafter.

## Model Construction

A two-dimensional data matrix with element *Y*_*ij*_ is considered, where examinees are indexed as *i* = 1,…,*N* and items are indexed as *j* = 1,…,*J*. If the *i*th examinee answers the *j*th item, the response is observed, and the *Y*_*ij*_ is equal to the observation *y*_*ij*_, otherwise, it is missing data. For convenience, the sign “*d*” is used to mark the missing data and the relevant parameters.

### Missing Data Model for Not-Reached Items

Glas and Pimentel (2008) proposed a sequential model with a linear restriction on the item parameters to model the not-reached items. Specifically, the missing indicator matrix **D** with element *d*_*ij*_ is given by:


(1) $$d_{ij}=\left\{ \begin{array}{cc} 0, & ify_{ij}wasobserverd,\\ 1, & ify_{ij}wasnotobserver. \end{array}\right.$$

where *d*_*ij*_ = 1indicates that the *i*th examinee drops out the *j*th item. Because of the small overall proportion of not-reached responses, the appropriate model must have few parameters to be estimable (Lord, 1983). The one-parameter logistic model (1PLM; Rasch, 1960) is adopted to model the missing indicators, thus the dropping-out probability of examinee *i* on item *j* is:


(2) $$p\left(d_{ij}=1|\theta_{i}^{d},\beta_{j}^{d}\right)=\frac{exp\left(\theta_{i}^{d}-\beta_{j}^{d}\right)}{1+exp\left(\theta_{i}^{d}-\beta_{j}^{d}\right)},$$

and


(3) $$\beta_{j}^{d}=\eta_{0}+\left(j-J\right)\eta_{1},$$

where $\beta_{j}^{d}$ represents the so-called item difficulty parameter for item *j*, and$\theta_{i}^{d}$ denotes the *i*th examinee’s dropping-out propensity. Also, $\beta_{j}^{d}=\eta_{0}$ when *j* = *J*, where η_0_ is the difficulty threshold of the last item, and η_1_ models a uniform change in the probability as a function of the item position in the test. Usually, the parameter η_1_ is negative, and hence it is more likely to drop out the test at later position items of the test.

### Higher-Order Deterministic Inputs, Noisy “And” Gate Model

The DINA model describes the probability of the item response as a function of latent attributes, and the probability of the *i*th examinee responding to item *j* correctly is as follows:


(4) p(Yij=1)=gj+(1-sj-gj)∏k=1Kαikqjk,

where *s_j_* and *g_j_* are the slipping and guessing probabilities of the *j*th item, respectively, 1−*s*_*j*_−*g*_*j*_ = *IDI*_*j*_ is the *j*th item discrimination index (de la Torre, 2008), and α_*ik*_ is the *k*th attribute of the *i*th examinee, with α_*ik*_ = 1 if examinee *i* masters attribute *k* and α_*ik*_ = 0 if examinee does not master attribute *k*. The Q matrix (Tatsuoka, 1983) is an *J*×*K* matrix, with *q*_*jk*_,*q_jk_* = 1 denoting that the attribute *k* is required for answering the *j*th item correctly and *q_jk_* = 0 if the attribute *k* is not required for answering the *j*th item correctly.

Equation (4) can be reparameterized as the reparameterized DINA model (DeCarlo, 2011).


(5) $$\beta_{j}=logit\left(g_{j}\right),$$

(6) $$\delta_{j}=logit\left(1-s_{j}\right)-logit\left(g_{j}\right).$$

In addition, $logit\left(x\right)=log\left(\frac{x}{1-x}\right),$ thus Equation (4) can be reformed as,


(7) $$logit\left(P\left(y_{ij}=1\right)\right)=\beta_{j}+\delta_{j}\prod_{k=1}^{K}\alpha_{ik}^{q_{jk}},$$

where β_*j*_ and δ_*j*_ are the item intercept and interaction parameter, respectively, and they are assumed to follow a bivariate normal distribution as follows:


(8) (βjδj)∼N((μβμδ),ΣI),ΣI=(σβ2σβδσβδσδ2).

The higher-order structure is very flexible because it can reduce the number of model parameters and can provide higher-order abilities and more accurate attribute structures. Because the attributes in a test are often correlated, the higher-order structure (de la Torre and Douglas, 2004; Zhan et al., 2018) for the attributes is expressed as,


(9) logit(P(αik=1))=θihγk-λk,

where *P*(α_*ik*_ = 1) is the probability that the *i*th examinee masters the *k*th attribute, $\theta_{i}^{h}$ is the higher-order ability of examinee *i*, and γ_*k*_ and λ_*k*_ are the slope and intercept parameters of attribute *k*, respectively. The slope parameter γ_*k*_ is positive because the knowledge attribute is mastered better with the increased ability $\theta {}_{i}^{h}$.

### Missing Mechanism Models

If the observation probability *p*(*y*_*ij*_|*d*_*ij*_, β_*j*_, δ_*j*_, α_*ik*_) does not depend on *θ*^**d**^, when *θ*^**h**^ and *θ*^**d**^ are independent, then the missing data are ignorable. In this situation, this model is treated as a MAR model. Let *p*(*y*_*ij*_|*d*_*ij*_, β_*j*_, δ_*j*_, α_*ik*_) be the measurement model for the observed data. In addition, let $p\left(d_{ij}|\theta_{i}^{d},\eta_{0},\eta_{1}\right)$ be the measurement model for the missing data indicators, and *p*(*θ*^**h**^) and *p*(*θ*^**d**^) are densities of *θ*^**h**^ and *θ*^**d**^, respectively. To model non-ignorable missing data, it is assumed that $\theta_{i}^{h}$ and $\theta_{i}^{d}$follow a bivariate normal distribution *N*(μ_P_, **Σ**_P_); thus, the two models describe the two missing mechanisms (i.e., MAR and NMAR). Next, we introduce the two missing data models for the not-reached items.

#### Missing at Random Model

The expression of the MAR model is as follows, and the likelihood function form of the MAR model can be written as,


(10) $$\prod_{i=1}^{N}\prod_{j=1}^{J}\prod_{k=1}^{K}p\left(\alpha_{ik}|\theta_{i}^{h},\gamma_{k},\lambda_{k}\right)p\left(d_{ij}|\theta_{i}^{d},\eta_{0},\eta_{1}\right)p\left(\theta_{i}^{h}\right)p\left(\theta_{i}^{d}\right),$$

where the MAR model is regarded as a model that ignores the missing data process. In fact, the latent variables $\theta_{i}^{h}$ and $\theta_{i}^{d}$ are independent in the MAR model. In other words, the model for the missing data process $p\left(d_{ij}|\theta_{i}^{d},\eta_{0},\eta_{1}\right)$ can be ignored in estimating the item response model.

#### Not Missing at Random Model

The NMAR model is often called the non-ignorable model, and in this case,$\theta_{i}^{h}$ and $\theta_{i}^{d}$ are correlated. A covariance matrix is used to describe the relationship between the latent higher-order ability parameters and the missing propensity parameters in this model. Thus, the likelihood function of the NMAR model can be written as,


(11) $$\prod_{i=1}^{N}\prod_{j=1}^{J}\prod_{k=1}^{K}p\left(\alpha_{ik}|\theta_{i}^{h},\gamma_{k},\lambda_{k}\right)p\left(d_{ij}|\theta_{i}^{d},\eta_{0},\eta_{1}\right)p\left(\theta_{i}^{h},\theta_{i}^{d}|\mu_{\text{P}},\Sigma_{\text{P}}\right),$$

where the person parameters are assumed to follow a bivariate normal distribution, with mean vector $\mu_{\text{P}}={\left(\mu_{\theta^{h}},\mu_{\theta^{d}}\right)}^{\prime }$ and covariance matrix:


(12) $$\Sigma_{\text{P}}=\left(\begin{array}{cc} \sigma_{\theta^{h}}^{2} & \sigma_{\theta^{h}\theta^{d}}\\ \sigma_{\theta^{h}\theta^{d}} & \sigma_{\theta^{d}}^{2} \end{array}\right).$$

### Model Identifications

In Equations (2) and (9), the linear parts of 1PLM and the HO-DINA model can be written as follows:


(13) $$\theta_{i}^{d}-\beta_{j}^{d}and\theta_{i}^{h}\gamma_{k}-\lambda_{k}.$$

To eliminate the trade-offs between ability $\theta_{i}^{d}$ and dropping-out threshold parameter $\beta_{j}^{d}$ and between the higher-order ability person parameter θ*^h^* and the attribute intercept λ_*k*_, the mean population level of person parameters is set to zero, that is, $\mu_{\theta^{h}}=0and\mu_{\theta^{d}}=0$. $\sigma_{\theta^{h}}=$ 1 is fixed to eliminate the scale trade-off between $\theta_{i}^{h}$ and γ_*k*_ (Lord and Novick, 1968; Fox, 2010). In addition to the identifications, two local independence assumptions are made, that is, the α_*ik*_ values are conditionally independent given $\theta_{i}^{h}$, and the *Y*_*ij*_ values are conditionally independent given α_*i*_.

### Bayesian Model Assessment

In the Bayesian framework, two common Bayesian model evaluation criteria, the deviance information criteria (DIC; Spiegelhalter et al., 2002) and the logarithm of the pseudo-marginal likelihood (LPML, Geisser and Eddy, 1979; Ibrahim et al., 2001) are used to compare the differences in the missing mechanism models according to the results of MCMC sampling. Let,


$$\Omega =\left\{\theta_{i}^{h},\theta_{i}^{d},\eta_{0},\eta_{1},\alpha_{ik},\beta_{j},\delta_{j},\gamma_{k},\lambda_{k},\mu_{\beta },\mu_{\delta },\Sigma_{I},\sigma_{\theta^{h}\theta^{d}},\sigma_{\theta^{d}}^{2}\right\}.$$

The DIC is given by,


(14) $${\text{Dev}}_{\left(Y,D|\Omega \right)}=-2logL\left(Y,D,\Omega \right)=-2\sum_{i=1}^{N}\sum_{j=1}^{J}\sum_{k=1}^{K}[\left(Y_{ij}=d\right)log\left(P\left(Y_{ij}=d\right)\right)+\left(Y_{ij}=1\right)log\left(\left(1-P\left(Y_{ij}=d\right)\right)P\left(Y_{ij}=1\right)\right)+\left(Y_{ij}=0\right)log\left(\left(1-P\left(Y_{ij}=d\right)\right)P\left(Y_{ij}=0\right)\right)].$$

On the basis of the posterior distribution of Dev(**Y**,**D**,**Ω**), the DIC was defined as,


(15) $$\text{DIC}=\bar{\text{Dev}}+p_{D}=\bar{\text{Dev}}+\left(\bar{\text{Dev}}-\hat{\text{Dev}}\right),$$

where $\bar{\text{Dev}}=E\left(\text{Dev}\left(Y,D,\Omega \right)|Y,D\right)≅\frac{1}{R}\sum_{r=1}^{R}\left[cpsbreak\right]\text{Dev}\left(Y,D,\Omega^{r}\right)$, which is the posterior mean deviance and is a Bayesian measure of fit, *r* = 1,…,*R* denotes the *r*th iteration of the algorithm, and $\hat{\text{Dev}}=\text{Dev}\left(Y,D,\bar{\Omega }\right)$, which is the effective number of parameters, is a Bayesian measure of complexity, with $\bar{\Omega }=E\left(\Omega |Y,D\right)≅\frac{1}{R}\sum_{r=1}^{R}\Omega^{r}$. A smaller DIC indicates a better model fit.

The conditional predictive ordinate (CPO) index of the two models was computed. Let *Q*_*ij*,*max*_ = *max*_1≤*r*≤*R*_{−log*f*(*Y*_*ij*,_*D*_*ij*_|Ω*^r^*)}. Thus,


(16) $$\text{log}\left(\hat{{\text{CPO}}_{ij}}\right)=-Q_{ij,max}-log\left[\frac{1}{R}\sum_{r=1}^{r}exp\left\{-logf\left(Y_{ij},D_{ij}|\Omega^{r}\right)-Q_{ij,max}\right\}\right].$$

The summary statistic for log$\left(\hat{{\text{CPO}}_{ij}}\right)$ is the sum of their logarithms, which is termed the LPML and is given by,


(17) $$\text{LPML}=\sum_{i=1}^{N}\sum_{j=1}^{J}\text{log}\left({\hat{\text{CPO}}}_{ij}\right),$$

where the model with a larger LPML indicates a better fit to the data.

## Simulation Studies

Three simulation studies were conducted to evaluate different aspects of the proposed model. Simulation study I was conducted to assess whether the MCMC algorithm could successfully recover parameters of the proposed model under different numbers of examinees and items. Simulation study II was conducted to investigate the parameter recovery of different numbers of attributes for the same examinees and items. Simulation study III intended to show the differences in model parameter estimates between the NMAR and MAR models for different dropping-out proportions and correlations among person parameters.

### Data Generation

In the three simulation studies, the item parameters were sampled from the following distributions: (β_*j*_δ_*j*_)∼*MVN*((μ_β_μ_δ_), **Σ_I_**), μ_β_ = −2.197,μ_δ_ = 4.394, **Σ_I_** = (1−0.8−0.81). These values were used in Shan and Wang (2020) study. The dropping-out proportions across three levels (i.e., low, medium, and high) were varied by setting different combinations of η_0_ and η_1_. That is, the dropping-out proportion was 3.8 (low) when η_0_ = 1,η_1_ = −0.7; the dropping-out proportion was 12 (medium) when η_0_ = 1,η_1_ = −0.32; and the dropping-out proportion was 25% (high) when η_0_ = 1,η_1_ = −0.18.

**FIGURE 1:**
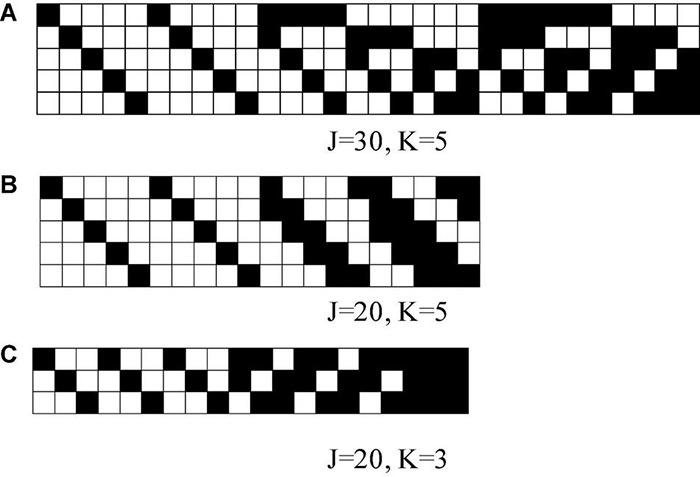
K-by-J Q matrices in simulation studies, where black means “1” and white means “0.” K is the number of attributes and J is the number of items.

The attribute intercept parameters were λ=(−1,−0.5, 0, 0.5, 1), and the attribute slope parameters wereγ_*k*_ = 1.5 for all attributes, which were consistent with those in the study by Shan and Wang (2020). Three Q matrices with different numbers of attributes (Figure 1) were considered, and the three Q matrices were taken from Xu and Shang (2018) study and Shan and Wang (2020) study.

The person parameters $\theta_{i}^{h}$ and $\theta_{i}^{d}$ were simulated from the bivariate normal distribution $\left(\begin{array}{c} \theta^{h}\\ \theta^{d} \end{array}\right)∼MVN\left(\left(\begin{array}{c} 0\\ 0 \end{array}\right),\left(\begin{array}{cc} 1 & \sigma_{\theta^{h}\theta^{d}}\\ \sigma_{\theta^{h}\theta^{d}} & \sigma_{\theta^{d}}^{2} \end{array}\right)\right),\text{where}\sigma_{\theta^{d}}^{2}=$ 0.25. Three levels of correlation between $\theta_{i}^{h}$ and $\theta_{i}^{d}$ were considered for $\rho_{\theta_{i}^{h}\theta_{i}^{d}}:$0 (uncorrelated), −0.5 (medium), and −0.8 (high). The missing data due to dropping-out items were simulated in the following manner. The three levels of dropping-out proportions were 3.8% (low), 12% (medium), and 25% (high).

### Model Calibration

The priors of η_0_ and η_1_ were η_0_ ∼ *N*(0,2) and η_1_ ∼ *N*(0,2), respectively. The priors of the item parameters β_*j*_ and δ_*j*_ were assumed to have a bivariate normal distribution: (β_*j*_δ_*j*_)∼*N*((μ_β_μ_δ_), **Σ_I_**). The priors of the person parameters were assumed to follow a bivariate normal distribution: (θ*^h^*θ*^d^*)∼*N*((00), **Σ_P_**). The priors of the higher-order structure parameters were expressed as λ_*k*_∼*N*(0,4) and γ_*k*_ ∼ *N*(0,4)*I*(γ_*k*_ > 0), the priors of the covariance matrix of the person were expressed as σ_θ*^h^*θ*^d^*_∼*U*(−1,1) and $\sigma_{\theta^{d}}^{2}$∼Inv-(2,2), the priors of the covariance matrix of the item parameters were expressed as **Σ_I_** Inv-Wishart $\left(\Sigma_{\mathrm{I0}}^{-1},v_{I0}\right)$, and the hyperpriors were specified as **Σ**_I0_ = (1001), *v*_*I*0_ = 2,*k*_*I*0_ = 1,μ_β_∼*N*(−2.197,2), and μ_δ_∼*N*(4.394,2)*I*(μ_δ_ > 0). The hyperpriors specified above were on a logit scale for β and δ and were consistent with those reported by Zhan et al. (2018). The mean guessing effect was set at 0.1, which was roughly equal to a logit value −2.197 for μ_β_. A standard deviation of $\sqrt{2}$ on the logit scale for μ_β_indicated that the simulated mean guessing effect changed from 0.026 to 0.314. In addition, the mean slipping effect was also set at 0.1, which indicated that μ_δ_ was approximately 4.394 on the logit scale. The simulated mean slipping effect changed from 0.007 to 0.653 under a standard deviation of $\sqrt{2}$ on the logit scale for δ.

The initial values of the model parameters were as follows: β_*j*_ = 0, δ_*j*_ = 0for *j* = 1,…,*J*,$\theta_{i}^{h}=0$, $\theta_{i}^{d}=0$for *i* = 1,…,*N*, σ_θ*^h^*θ*^d^*_ = 0, $\sigma_{\theta^{d}}^{2}=1$, η_0_ = 0,η_1_ = 0,μ_β_ = 0,μ_δ_ = 0, **Σ_P_** = (1001), μ_**P**_ = (00), and $\sigma_{\theta^{h}}^{2}=1$. In addition, λ_*k*_ = 0,γ_*k*_ = 1for *k* = 1,,*K*, and α=(α_11_⋯α_1*K*_⋮⋱⋮α_*N*1_⋯α_*NK*_), where α_*ik*_ (*i* = 1,…,*N*,*k* = 1,…,*K*) were sampled from 0 to 1 randomly. The proposal variances were chosen to give Metropolis acceptance rates between 25% and 40%. The Markov chain length was set at 10,000 so that the potential scale reduction factor (PSRF; Brooks and Gelman, 1998) was less than 1.1 for all parameters, which implied proper chain convergence. Five thousand iterations were treated as burn-in. The final parameter estimates were obtained as the average of the post-burn-in iterations.

In terms of evaluation criteria, the bias and root mean squared error (RMSE) are used to assess the accuracy of the parameter estimates. In particular, the bias for parameter η was,


(18) $$\text{bias}\left(\eta \right)=\frac{1}{R}\sum_{r=1}^{R}\left({\hat{\eta }}^{\left(r\right)}-\eta \right),$$

and the RMSE for parameter η is defined as,


(19) $$\text{RMSE}\left(\eta \right)=\sqrt{\frac{1}{R}\sum_{r=1}^{R}{\left({\hat{\eta }}^{\left(r\right)}-\eta \right)}^{2}},$$

where η is the true value of the parameter, and ${\hat{\eta }}^{\left(r\right)}$ is the estimate for the *r*th replication. There were *R* = 30 replications for each simulation condition. The recoveries of attributes are evaluated using the attribute correct classification rate (ACCR) and the pattern correct classification rate (PCCR):


(20) $$\text{ACCR}=\frac{\sum_{i=1}^{N}I\left(\hat{\alpha_{ik}}=\alpha_{ik}\right)}{N},$$

(21) $$\text{PCCR}=\frac{\sum_{i=1}^{N}\left[\prod_{k=1}^{K}I\left(\hat{\alpha_{ik}}=\alpha_{ik}\right)\right]}{N},$$

where $I\left(\hat{\alpha_{ik}}=\alpha_{ik}\right)$ is the indicator function that is, $I\left(\hat{\alpha_{ik}}=\alpha_{ik}\right)=1$ if $\hat{\alpha_{ik}}=\alpha_{ik}$, otherwise $I\left(\hat{\alpha_{ik}}=\alpha_{ik}\right)=0$.

### Simulation Study I

In simulation study I, the different numbers of examinees and items were considered to estimate the model parameters under a fixed number of five attributes. Three conditions were considered in this simulation: (a) 500 examinees and 30 items, (b) 1,000 examinees and 30 items, and (c) 500 examinees and 20 items. The correlation between $\theta_{i}^{h}$ and $\theta_{i}^{d}$ was −0.3, and the dropping-out proportion was medium.

**FIGURE 2:**
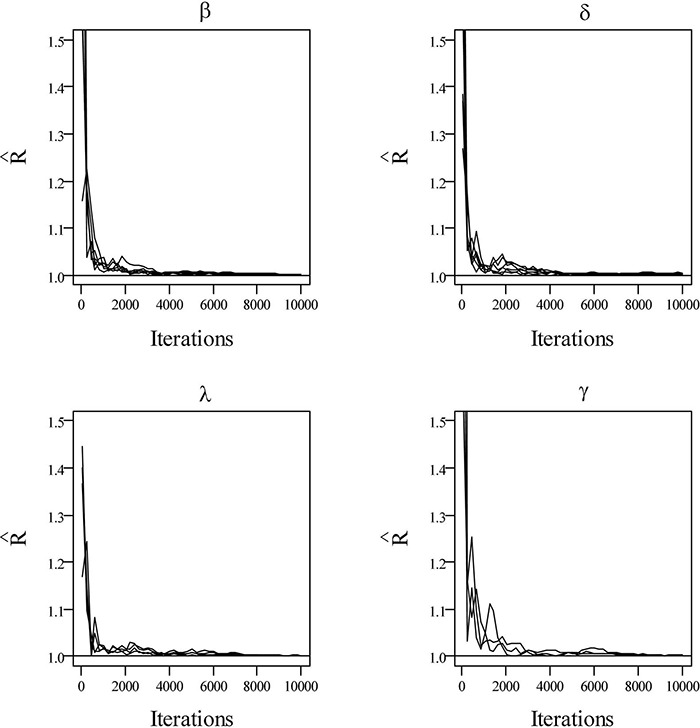
The trace plots of PSRF values for simulation study I.

Table 1 presents the bias and RMSE of the ability parameters and item parameters, as well as the attribute parameter estimates. For the 30 items and the 5 attributes (please see the first four columns of Table 1), the item parameter estimates improve when the number of examinees increases from 500 to 1,000, the bias and RMSE of δ and μ_β_decrease, and the RMSE of β,μ_δ_, and item covariance matrix elements reduce. For the 500 examinees and the 5 attributes (please see the middle four columns of Table 1), the person parameter estimates improve when the number of items increases from 20 to 30, and *θ*^**h**^ and $\sigma_{\theta^{d}}^{2}$ are more accurate. The ACCRs and PCCRs are presented in Table 2. The ACCRs and PCCRs could be recovered satisfactorily with a larger sample and longer test length. The ACCRs and PCCRs decrease when the number of examinees or test length decreases (please see the first three columns in Table 2), and the changes are particularly marked when the test length is reduced. Figure 2 shows the PSRF of several items and attribute parameters under 500 examinees and 30 items. It is observed that the item intercept parameter β, the interaction parameter δ, the attribute slope parameter γ, and the attribute intercept parameter λ converge at 5,000 iterations, and the convergence of β and δ are significantly faster than that of λ and γ.

**TABLE 1:** Bias and RMSE of the parameter estimates in simulation studies I and II.

	*N* = 1000		*N* = 500		*N* = 500		*N* = 500	
	*J* = 30		*J* = 30		*J* = 20		*J* = 20	
	*K* = 5		*K* = 5		*K* = 5		*K* = 3	
Parameter	Bias	RMSE	Bias	RMSE	Bias	RMSE	Bias	RMSE
β	0.009	**0.167**	−0.002	**0.198**	−0.134	0.272	−0.020	0.282
δ	**−0.001**	**0.274**	**−0.051**	**0.339**	0.072	0.345	0.017	0.351
μ_β_	**−0.111**	**0.203**	**−0.120**	**0.215**	−0.296	0.374	−0.192	0.268
μ_δ_	0.035	**0.181**	−0.017	**0.196**	0.236	0.356	0.191	0.313
λ_1_	0.078	0.137	0.063	0.179	0.066	0.191	−0.109	0.181
λ_2_	0.029	0.100	−0.133	0.193	−0.149	0.199	−0.030	0.130
λ_3_	0.052	0.104	−0.058	0.143	−0.127	0.202	−0.204	0.245
λ_4_	0.040	0.106	−0.069	0.145	−0.121	0.178	−	−
λ_5_	0.201	0.239	−0.089	0.188	−0.181	0.246	−	−
γ_1_	0.129	0.249	0.296	0.457	0.222	0.403	−0.179	0.451
γ_2_	0.034	0.189	0.065	0.268	−0.288	0.360	−0.156	0.545
γ_3_	−0.063	0.182	−0.002	0.252	0.359	0.527	−0.301	0.626
γ_4_	−0.027	0.180	−0.202	0.298	−0.139	0.276	−	−
γ_5_	0.039	0.206	0.153	0.326	−0.083	0.282	−	−
$\sigma_{\beta }^{2}$	−0.152	**0.281**	−0.051	**0.282**	−0.035	0.374	−0.353	0.429
σ_βδ_	0.093	**0.244**	−0.027	**0.280**	−0.118	0.415	0.131	0.318
$\sigma_{\delta }^{2}$	−0.103	**0.282**	0.066	**0.340**	0.315	0.611	0.132	0.457
η_0_	−0.051	**0.086**	**−0.014**	**0.097**	0.053	0.112	−0.130	0.161
η_1_	**−0.004**	**0.013**	**0.005**	**0.017**	0.008	0.019	−0.013	0.022
σ_θ*^h^*θ*^d^*_	−0.056	0.077	−0.046	0.091	0.001	0.083	0.057	0.105
$\sigma_{\theta^{d}}^{2}$	−0.001	0.081	**0.008**	**0.094**	**0.018**	**0.101**	−0.029	0.075
θ*^h^*	0.071	0.625	**−0.043**	**0.594**	**−0.044**	**0.612**	−0.044	0.701
θ*^d^*	−0.039	0.480	0.006	0.475	0.006	0.468	0.006	0.479

*The boldfaced values indicate that much smaller Bias and RMSE are obtained from the model.*

**TABLE 2:** ACCRs and PCCRs in simulation studies I and II.

	*N* = 1000	*N* = 500	*N* = 500	*N* = 500
	*J* = 30	*J* = 30	*J* = 20	*J* = 20
	*K* = 5	*K* = 5	*K* = 5	*K* = 3
ACCR	0.968	0.966	0.922	0.985
	0.980	0.976	0.966	0.993
	0.984	0.985	0.960	0.982
	0.986	0.977	0.984	−
	0.986	0.981	0.954	−
PCCR	**0.910**	**0.898**	**0.811**	**0.961**

*The boldfaced values indicate that much smaller Bias and RMSE are obtained from the model.*

### Simulation Study II

This simulation study was conducted to investigate the parameter recovery of different numbers of attributes for fixed 500 examinees and 20 items. The correlation between $\theta_{i}^{h}$ and $\theta_{i}^{d}$ was set at −0.3, and the dropping-out proportion was medium.

The last four columns of Table 1 show the results of simulation study II. The RMSE of the estimates of item and person parameters with attribute *K* = 5 are smaller than those with attribute *K* = 3. The RMSE of the attribute slope parameters and intercept parameters recover more satisfactorily with attribute *K* = 3 than with attribute *K* = 5. The last two columns of Table 2 show the ACCRs and PCCRs for simulation study II. The ACCRs with attribute *K* = 3 are higher than those with attribute *K* = 5 and improve from 0.957 to 0.987 on average. Moreover, the PCCRs are significantly higher when the number of attributes decreases. That is, the PCCR with attribute *K* = 5 is 0.811, and the PCCR with attribute *K* = 3 is 0.961.

### Simulation Study III

The purpose of this simulation study was to investigate the parameter recovery with the NMAR model, MAR model, and HO-DINA model that ignores the not-reached items under different simulation conditions. The data were generated using the proposed model with the NMAR mechanism. A total of 500 examinees answered 30 items, and each item had 5 attributes. Three dropping-out proportions (i.e., 3.8% [low], 12% [medium], and 25% [high]) and three correlations between $\theta_{i}^{h}$ and $\theta_{i}^{d}$ (i.e., 0 [uncorrelated], −0.5 [medium], and −0.8 [high]) were manipulated. Thus, there were 3 × 3 simulation conditions.

**FIGURE 3:**
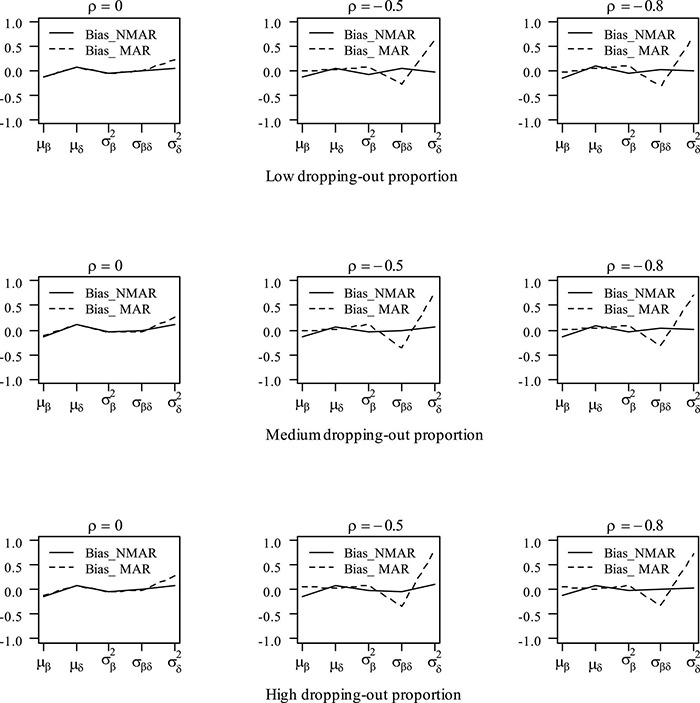
Bias of parameter estimates in the mean item vector and the item covariance matrix elements under different dropping-out proportions and correlations between $\theta_{i}^{h}$ and $\theta_{i}^{d}$ in simulation study III. Note that the Bias_NMAR is the bias of parameter estimates in NMAR model, and Bias_MAR is the bias of parameter estimates in the MAR model.

**FIGURE 4:**
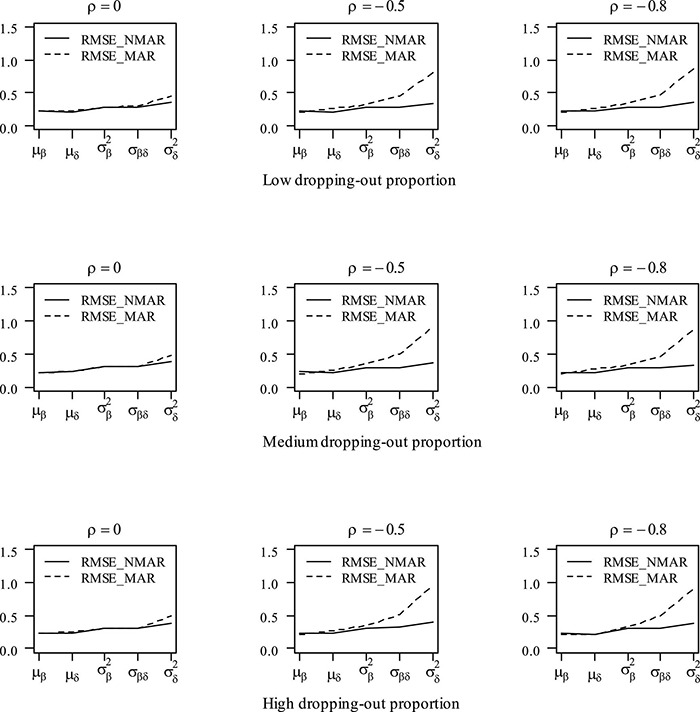
RMSE of parameter estimates in the mean item vector and the item covariance matrix elements under different dropping-out proportions and correlations between $\theta_{i}^{h}$ and $\theta_{i}^{d}$ in simulation study III. Note that the Bias_NMAR is the bias of parameter estimates in the NMAR model, and Bias_MAR is the bias of parameter estimates in the MAR model.

**FIGURE 5:**
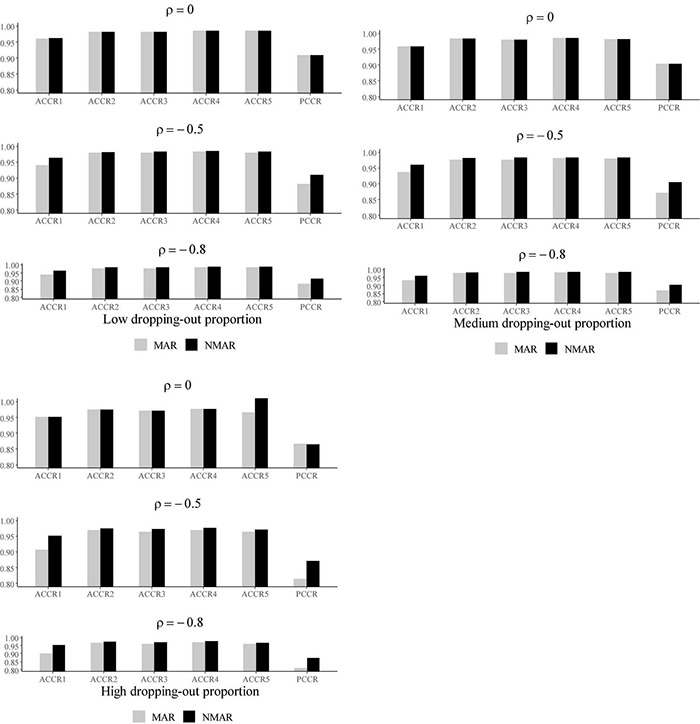
The ACCRs and PCCRs of NMAR and MAR models under different correlations between $\theta_{i}^{h}$ and $\theta_{i}^{d}$ and different dropping-out proportions in simulation study III.

Table 3 shows the bias and RMSE of the parameters of three models with low dropping-out proportions under different correlations between $\theta_{i}^{h}$ and $\theta_{i}^{d}$. Results show that the parameter estimates from the three models are similar when the correlation between $\theta_{i}^{h}$ and $\theta_{i}^{d}$ is 0. When the correlation between $\theta_{i}^{h}$ and $\theta_{i}^{d}$ increases, the bias and RMSE of η_1_, β, **Σ_I_**, and γ in the NMAR model are much smaller than those in the MAR and HO-DINA models. Moreover, for low dropping-out proportions, when the correlation between $\theta_{i}^{h}$ and $\theta_{i}^{d}$ increases, the bias of the person parameters of the three models changes very little, whereas the RMSE of the person parameters in the MAR and HO-DINA models increases significantly. As expected, the NMAR model has higher accuracy of parameters than that of the other two models. Furthermore, the parameter estimates of the MAR and HO-DINA models are similar for all simulation conditions because $\theta_{i}^{h}$ and $\theta_{i}^{d}$ are uncorrelated in both the MAR and HO-DINA models, which ignore the not-reached items. Table 4 shows the bias and RMSE of the parameters of the three models with medium dropping-out proportions under different correlations between $\theta_{i}^{h}$ and $\theta_{i}^{d}$. Similar parameter estimates are obtained from the three models when the correlation between $\theta_{i}^{h}$ and $\theta_{i}^{d}$ is 0. When the correlation between $\theta_{i}^{h}$ and $\theta_{i}^{d}$ increases, not only the bias but also the RMSE of the person parameters are lower in the NMAR model than those in the MAR and HO-DINA models, and the other results are similar to those with low dropping-out proportions. Table 5 shows the bias and RMSE of the parameters of the three models with high dropping-out proportions under different correlations between $\theta_{i}^{h}$ and $\theta_{i}^{d}$. We find that the parameter estimates improve significantly with high dropping-out proportions. Figure 3 shows the bias of the estimates of item mean vector and the item covariance matrix elements in the NMAR and MAR models under different dropping-out proportions and correlations between $\theta_{i}^{h}$ and $\theta_{i}^{d}$. The results show that the estimates of the parameters are more accurate in the NMAR model than those in the MAR model when the correlation is increased. Moreover, it is observed that the bias of the parameters of the NMAR model is close to 0 as the correlation between $\theta_{i}^{h}$ and $\theta_{i}^{d}$ increases. In contrast, the bias of the parameters of the MAR model is significantly larger than that of the NMAR model. Figure 4 shows the RMSE of the estimates of the item mean vector and the item covariance matrix elements in the NMAR and MAR models under different dropping-out proportions and correlations between $\theta_{i}^{h}$ and $\theta_{i}^{d}$. The results show that the RMSE of the item mean vector in the NMAR model improves slightly than that in the MAR model. Moreover, the RMSE of the item covariance matrix elements shows significant improvements, and the estimates of the item covariance matrix elements are precise when the correlation is high. Figure 5 shows the ACCRs and PCCRs under nine simulation conditions. Detailed results are provided in Supplementary Table 1. It is found that ACCRs and PCCRs in the NMAR model are improved significantly when the missing proportion or the correlation between $\theta_{i}^{h}$ and $\theta_{i}^{d}$ is high. This indicates that the MAR model could not recover the attribute pattern effectively when the missing data mechanism is indeed non-ignorable. Table 6 shows the model selection results. The differences in DIC and LPML are not obvious when the correlation between $\theta_{i}^{h}$ and $\theta_{i}^{d}$ is 0. The DICs of the NMAR model are smaller than those of the MAR model under nine simulation conditions. Moreover, the LPMLs of the NMAR model are higher than those of the MAR model. Thus, the DIC and LPML indices are able to select the true model accurately.

**TABLE 3:** Bias and RMSE of parameter estimates of three models with low dropping-out proportion under different correlations between $\theta_{i}^{h}$ and $\theta_{i}^{d}$ in simulation study III.

		ρ=0			ρ=−0.5			ρ=−0.8		
Parameter	Statistics	NMAR	MAR	HO-DINA	NMAR	MAR	HO-DINA	NMAR	MAR	HO-DINA
η_0_	Bias	0.003	0.001	−	0.036	−0.001	−	**0.015**	**−0.019**	−
	RMSE	0.123	0.125	−	**0.155**	**0.174**	−	**0.134**	**0.162**	−
η_1_	Bias	0.005	0.004	−	**−0.004**	**−0.109**	−	**−0.003**	**−0.107**	−
	RMSE	0.055	0.055	−	**0.065**	**0.137**	−	**0.059**	**0.131**	−
β	Bias	−0.018	−0.016	−0.015	**−0.003**	**0.124**	0.121	**−0.029**	**0.093**	0.093
	RMSE	0.234	0.233	0.234	**0.239**	**0.299**	0.297	**0.237**	**0.285**	0.286
δ	Bias	0.039	0.047	0.045	0.022	−0.017	−0.015	0.063	0.021	0.021
	RMSE	0.336	0.345	0.346	**0.341**	**0.369**	0.369	**0.346**	**0.369**	0.369
μ_β_	Bias	−0.136	−0.117	−0.115	−0.120	0.006	0.004	−0.146	−0.022	−0.022
	RMSE	0.228	0.217	0.218	0.218	0.201	0.201	0.235	0.204	0.204
μ_δ_	Bias	0.073	0.067	0.064	0.054	0.016	0.017	0.095	0.052	0.052
	RMSE	0.216	0.228	0.229	**0.205**	**0.255**	0.255	**0.223**	**0.259**	0.263
$\sigma_{\beta }^{2}$	Bias	−0.052	−0.053	−0.056	**−0.067**	**0.074**	0.075	**−0.055**	**0.096**	0.096
	RMSE	0.290	0.290	0.289	**0.291**	**0.322**	0.322	**0.291**	**0.331**	0.332
σ_βδ_	Bias	0.008	−0.005	−0.003	**0.051**	**−0.275**	−0.276	**0.028**	**−0.316**	−0.314
	RMSE	0.286	0.299	0.296	**0.281**	**0.446**	0.445	**0.289**	**0.479**	0.478
$\sigma_{\delta }^{2}$	Bias	0.054	0.225	0.222	**−0.021**	**0.656**	0.657	**0.004**	**0.703**	0.700
	RMSE	0.358	0.447	0.443	**0.333**	**0.812**	0.811	**0.355**	**0.856**	0.855
λ_**1**_	Bias	0.039	0.017	0.017	**0.098**	**0.298**	0.285	**0.056**	**0.220**	0.224
	RMSE	0.168	0.172	0.173	**0.193**	**0.370**	0.363	**0.181**	**0.331**	0.330
λ_**2**_	Bias	−0.096	−0.111	−0.108	−0.103	−0.051	−0.055	−0.096	−0.049	−0.048
	RMSE	0.168	0.180	0.178	0.168	0.160	0.163	0.166	0.159	0.159
λ_**3**_	Bias	−0.051	−0.053	−0.052	−0.127	−0.003	−0.011	−0.091	0.030	0.033
	RMSE	0.147	0.149	0.150	0.188	0.163	0.162	**0.167**	**0.169**	0.168
λ_**4**_	Bias	−0.089	−0.084	−0.083	**−0.068**	**0.023**	0.018	−0.080	0.002	0.003
	RMSE	0.162	0.161	0.161	0.152	0.149	0.150	0.153	0.141	0.141
λ_**5**_	Bias	−0.102	−0.076	−0.081	−0.142	0.019	0.017	−0.135	0.006	0.007
	RMSE	0.194	0.186	0.190	0.214	0.185	0.187	0.210	0.181	0.180
γ_**1**_	Bias	0.122	0.173	0.179	**0.178**	**0.294**	0.263	**0.277**	**0.501**	0.520
	RMSE	0.346	0.371	0.374	**0.387**	**0.472**	0.433	**0.451**	**0.698**	0.710
γ_**2**_	Bias	−0.004	0.044	0.035	**−0.117**	**0.246**	0.245	**−0.084**	**0.246**	0.247
	RMSE	0.276	0.284	0.275	**0.281**	**0.380**	0.381	**0.271**	**0.372**	0.377
γ_**3**_	Bias	0.080	0.104	0.111	**0.126**	**0.474**	0.477	**0.141**	**0.485**	0.494
	RMSE	0.301	0.312	0.313	**0.323**	**0.577**	0.583	**0.332**	**0.594**	0.603
γ_**4**_	Bias	−0.103	−0.077	−0.078	−0.114	0.025	0.021	−0.178	−0.037	−0.035
	RMSE	0.267	0.263	0.264	0.274	0.252	0.256	0.287	0.235	0.233
γ_**5**_	Bias	−0.052	0.005	−0.006	**−0.075**	**0.114**	0.115	**−0.039**	**0.137**	0.132
	RMSE	0.289	0.286	0.290	**0.284**	**0.307**	0.310	**0.280**	**0.313**	0.309
*θ* ^**d**^	Bias	−0.002	−0.002	−	**0.011**	**0.011**	−	**0.017**	**0.018**	−
	RMSE	0.499	0.492	−	**0.454**	**0.667**	−	**0.377**	**0.668**	−
*θ* ^**h**^	Bias	−0.044	−0.046	−0.046	**−0.044**	**−0.044**	−0.047	**−0.044**	**−0.046**	−0.045
	RMSE	0.581	0.581	0.580	**0.582**	**0.591**	0.592	**0.578**	**0.591**	0.591
$\sigma_{\theta^{d}}^{2}$	Bias	−0.002	0.007	−	**0.013**	**1.022**	−	**0.015**	**1.023**	−
	RMSE	0.089	0.088	−	**0.095**	**1.160**	−	**0.081**	**1.097**	−
σ_*θ*^**h**^*θ*_**d**__	Bias	0.011	−	−	0.015	−	−	0.010	−	−
	RMSE	0.131	−	−	0.113	−	−	0.082	−	−

*NMAR means not missing at random model, MAR means missing at random model, HO-DINA means higher-order DINA model. The boldfaced values indicate that much smaller Bias and RMSE are obtained from the model.*

**TABLE 4:** Bias and RMSE of parameter estimates of three models with medium dropping-out proportion under different correlations between $\theta_{i}^{h}$ and $\theta_{i}^{d}$ in simulation study III.

		ρ=0			ρ=−0.5			ρ=−0.8		
Parameter	Statistics	NMAR	MAR	HO-DINA	NMAR	MAR	HO-DINA	NMAR	MAR	HO-DINA
η_0_	Bias	0.014	0.009	−	**−0.006**	**−0.159**	−	**−0.033**	**−0.181**	−
	RMSE	0.133	0.131	−	**0.131**	**0.216**	−	**0.123**	**0.226**	−
η_1_	Bias	0.001	0.001	−	**−0.001**	**−0.039**	−	**−0.011**	**−0.048**	−
	RMSE	−0.002	−0.003	−	**−0.001**	**−0.024**	−	**−0.001**	**−0.019**	−
β	Bias	−0.022	−0.019	−0.019	**−0.028**	**0.114**	0.113	−0.021	0.119	0.118
	RMSE	0.249	0.248	0.249	**0.265**	**0.323**	0.322	**0.249**	**0.309**	0.309
δ	Bias	0.071	0.082	0.081	0.042	−0.002	−0.001	0.052	0.005	0.007
	RMSE	0.365	0.378	0.377	**0.360**	**0.401**	0.400	**0.357**	**0.389**	0.391
μ_β_	Bias	−0.137	−0.121	−0.120	−0.146	−0.001	0.001	−0.134	0.008	0.004
	RMSE	0.229	0.226	0.223	0.238	0.206	0.204	0.229	0.207	0.202
μ_δ_	Bias	0.102	0.103	0.102	0.077	0.029	0.026	0.080	0.032	0.037
	RMSE	0.232	0.250	0.247	**0.224**	**0.266**	0.264	**0.226**	**0.269**	0.268
$\sigma_{\beta }^{2}$	Bias	−0.031	−0.031	−0.033	**−0.032**	**0.105**	0.108	**−0.046**	**0.095**	0.095
	RMSE	0.308	0.307	0.306	**0.299**	**0.341**	0.342	**0.299**	**0.338**	0.338
σ_βδ_	Bias	−0.015	−0.024	−0.023	**−0.015**	**−0.344**	−0.346	**0.029**	**−0.304**	−0.304
	RMSE	0.310	0.319	0.319	**0.296**	**0.504**	0.505	**0.286**	**0.471**	0.471
$\sigma_{\delta }^{2}$	Bias	0.107	0.277	0.274	**0.075**	**0.764**	0.765	**0.016**	**0.710**	0.712
	RMSE	0.393	0.490	0.488	**0.361**	**0.919**	0.919	**0.340**	**0.864**	0.866
λ_**1**_	Bias	0.047	0.026	0.028	**0.109**	**0.349**	0.344	**0.070**	**0.267**	0.268
	RMSE	0.170	0.173	0.172	**0.195**	**0.414**	0.410	**0.187**	**0.375**	0.372
λ_**2**_	Bias	−0.104	−0.116	−0.114	−0.106	−0.055	−0.052	−0.110	−0.051	−0.048
	RMSE	0.174	0.184	0.182	0.171	0.163	0.163	0.173	0.158	0.157
λ_**3**_	Bias	−0.044	−0.047	−0.044	−0.112	0.025	0.032	−0.097	0.027	0.026
	RMSE	0.146	0.148	0.150	0.180	0.171	0.180	0.168	0.165	0.161
λ_**4**_	Bias	−0.091	−0.086	−0.083	−0.064	0.034	0.034	−0.081	0.011	0.009
	RMSE	0.165	0.162	0.162	**0.152**	**0.154**	0.155	0.156	0.144	0.144
λ_**5**_	Bias	−0.107	−0.083	−0.082	−0.153	0.003	0.005	−0.138	0.033	0.037
	RMSE	0.197	0.194	0.192	0.221	0.182	0.181	0.214	0.190	0.191
γ_**1**_	Bias	0.119	0.183	0.168	**0.113**	**0.301**	0.285	**0.236**	**0.723**	0.712
	RMSE	0.170	0.173	0.172	**0.195**	**0.414**	0.410	**0.187**	**0.375**	0.372
γ_**2**_	Bias	−0.006	0.032	0.029	**−0.110**	**0.267**	0.269	**−0.098**	**0.233**	0.232
	RMSE	0.268	0.277	0.271	**0.280**	**0.393**	0.398	**0.274**	**0.365**	0.367
γ_**3**_	Bias	0.096	0.104	0.124	**0.127**	**0.504**	0.516	**0.127**	**0.473**	0.472
	RMSE	0.313	0.307	0.322	**0.332**	**0.611**	0.632	**0.323**	**0.580**	0.578
γ_**4**_	Bias	−0.122	−0.093	−0.089	−0.091	0.056	0.046	−0.176	−0.046	−0.054
	RMSE	0.277	0.269	0.264	0.267	0.265	0.263	0.295	0.240	0.237
γ_**5**_	Bias	−0.059	−0.006	−0.011	**−0.079**	**0.087**	0.084	**−0.045**	**0.152**	0.161
	RMSE	0.284	0.298	0.285	**0.285**	**0.293**	0.289	**0.284**	**0.325**	0.333
*θ* ^**d**^	Bias	−0.002	−0.002	−	**0.011**	**0.013**	−	**0.017**	**0.019**	−
	RMSE	0.484	0.483	−	**0.443**	**0.577**	−	**0.379**	**0.586**	−
*θ* ^**h**^	Bias	−0.044	−0.045	−0.045	**−0.044**	**−0.047**	−0.045	**−0.044**	**−0.045**	−0.045
	RMSE	0.585	0.583	0.583	**0.581**	**0.593**	0.592	**0.574**	**0.592**	0.593
$\sigma_{\theta^{d}}^{2}$	Bias	0.008	0.011	−	**0.013**	**0.598**	−	**0.051**	**0.648**	−
	RMSE	0.001	0.001	−	**0.017**	**0.494**	−	**0.029**	**0.411**	−
σ_*θ*^**h**^*θ*^**d**^_	Bias	−0.003	−	−	0.008	−	−	0.001	−	−
	RMSE	0.023	−	−	0.005	−	−	0.008	−	−

*The boldfaced values indicate that much smaller Bias and RMSE are obtained from the model.*

**TABLE 5:** Bias and RMSE of parameter estimates of three models with high dropping-out proportion under different correlations between $\theta_{i}^{h}$ and $\theta_{i}^{d}$ in simulation study III.

		ρ=0			ρ=−0.5			ρ=−0.8		
Parameter	Statistics	NMAR	MAR	HO-DINA	NMAR	MAR	HO-DINA	NMAR	MAR	HO-DINA
η_0_	Bias	−0.013	−0.019	−	**0.016**	**−0.221**	−	**0.014**	**−0.174**	−
	RMSE	0.134	0.132	−	**0.146**	**0.271**	−	**0.130**	**0.237**	−
η_1_	Bias	−0.002	−0.003	−	**−0.001**	**−0.024**	−	**−0.001**	**−0.019**	−
	RMSE	0.012	0.011	−	**0.013**	**0.028**	−	**0.011**	**0.024**	−
β	Bias	−0.025	−0.021	−0.021	**−0.027**	**0.175**	0.177	**−0.010**	**0.187**	0.185
	RMSE	0.275	0.274	0.273	**0.284**	**0.383**	0.384	**0.267**	**0.373**	0.371
δ	Bias	0.058	0.071	0.069	0.060	−0.001	−0.003	0.043	−0.021	−0.019
	RMSE	0.392	0.405	0.404	**0.392**	**0.441**	0.443	**0.378**	**0.427**	0.425
μ_β_	Bias	−0.142	−0.120	−0.124	−0.144	0.056	0.061	−0.126	0.071	0.067
	RMSE	0.235	0.225	0.228	0.240	0.217	0.218	0.227	0.216	0.215
μ_δ_	Bias	0.091	0.089	0.092	0.093	0.035	0.028	0.075	0.011	0.015
	RMSE	0.234	0.252	0.253	**0.236**	**0.271**	0.272	**0.219**	**0.263**	0.216
$\sigma_{\beta }^{2}$	Bias	−0.032	−0.033	−0.032	**−0.012**	**0.084**	0.086	**−0.025**	**0.084**	0.085
	RMSE	0.302	0.302	0.302	**0.313**	**0.339**	0.339	**0.304**	**0.332**	0.334
σ_βδ_	Bias	−0.004	−0.013	−0.017	**−0.047**	**−0.336**	−0.339	**−0.004**	**−0.313**	−0.314
	RMSE	0.302	0.316	0.314	**0.322**	**0.505**	0.507	**0.309**	**0.485**	0.486
$\sigma_{\delta }^{2}$	Bias	0.083	0.271	0.271	**0.118**	**0.802**	0.806	**0.037**	**0.738**	0.740
	RMSE	0.383	0.491	0.489	**0.405**	**0.960**	0.965	**0.378**	**0.898**	0.901
λ_**1**_	Bias	0.047	0.027	0.021	**0.110**	**0.490**	0.502	**0.089**	**0.474**	0.467
	RMSE	0.182	0.181	0.184	**0.201**	**0.553**	0.566	**0.198**	**0.552**	0.544
λ_**2**_	Bias	−0.110	−0.120	−0.122	−0.099	0.013	0.015	−0.102	0.006	0.003
	RMSE	0.182	0.190	0.191	**0.170**	**0.173**	0.174	0.174	0.164	0.163
λ_**3**_	Bias	−0.055	−0.055	−0.055	−0.116	0.102	0.104	−**0.091**	**0.152**	0.144
	RMSE	0.156	0.158	0.156	**0.186**	**0.206**	0.207	**0.171**	**0.237**	0.232
λ_**4**_	Bias	−0.096	−0.089	−0.092	**−0.074**	**0.098**	0.098	**−0.076**	**0.085**	0.084
	RMSE	0.170	0.167	0.168	**0.160**	**0.188**	0.188	**0.159**	**0.178**	0.177
λ_**5**_	Bias	−0.077	−0.045	−0.051	−0.147	0.141	0.147	**−0.140**	**0.171**	0.164
	RMSE	0.196	0.197	0.195	**0.223**	**0.244**	0.247	**0.225**	**0.269**	0.263
γ_**1**_	Bias	−0.133	0.174	0.186	**0.147**	**0.672**	0.720	**0.251**	**1.029**	0.995
	RMSE	0.374	0.390	0.400	**0.375**	**0.892**	0.952	**0.439**	**1.294**	1.249
γ_**2**_	Bias	−0.020	0.059	0.058	**−0.102**	**0.334**	0.340	**−0.058**	**0.341**	0.333
	RMSE	0.287	0.302	0.294	**0.285**	**0.458**	0.461	**0.271**	**0.452**	0.444
γ_**3**_	Bias	−0.091	0.117	0.117	**0.111**	**0.537**	0.532	**0.143**	**0.591**	0.584
	RMSE	0.328	0.330	0.328	**0.332**	**0.648**	0.644	**0.341**	**0.700**	0.693
γ_**4**_	Bias	−0.130	−0.104	−0.102	−0.122	0.055	0.053	−0.146	0.026	0.026
	RMSE	0.289	0.280	0.277	0.290	0.271	0.270	0.294	0.265	0.261
γ_**5**_	Bias	−0.014	0.046	0.039	**−0.078**	**0.147**	0.152	**−0.050**	**0.226**	0.213
	RMSE	0.304	0.321	0.314	**0.289**	**0.330**	0.334	**0.296**	**0.386**	0.376
*θ* ^**d**^	Bias	−0.002	−0.002	−	**0.011**	**0.016**	−	**0.018**	**0.021**	−
	RMSE	0.479	0.475	−	**0.442**	**0.549**	−	**0.374**	**0.526**	−
*θ* ^**h**^	Bias	−0.044	−0.045	−0.046	**−0.043**	**−0.046**	−0.045	**−0.044**	**−0.045**	−0.046
	RMSE	0.595	0.594	0.594	**0.590**	**0.607**	0.608	**0.584**	**0.607**	0.607
$\sigma_{\theta^{d}}^{2}$	Bias	0.001	0.001	−	**0.017**	**0.494**	−	**0.029**	**0.411**	−
	RMSE	0.088	0.085	−	**0.102**	**0.555**	−	**0.097**	**0.463**	−
σ_*θ*^**h**^*θ*^**d**^_	Bias	0.023	−	−	0.005	−	−	0.008	−	−
	RMSE	0.102	−	−	0.092	−	−	0.085	−	−

*The boldfaced values indicate that much smaller Bias and RMSE are obtained from the model.*

**TABLE 6:** DICs and LPMLs of NMAR and MAR models under different correlations between $\theta_{i}^{h}$ and $\theta_{i}^{d}$ and different dropping-out proportions in simulation study III.

		Low dropping-out proportion		Medium dropping-out proportion		High dropping-out proportion	
		NMAR	MAR	NMAR	MAR	NMAR	MAR
ρ=0	DIC	12139.3	12146.3	12283.9	12290.6	12084.8	12090.3
	LPML	−6348.4	−6352.7	−6465.8	−6468.3	−6532.1	−6539.9
ρ=−0.5	DIC	12152.6	12541.4	12225.5	12653.3	12113.8	12570.5
	LPML	−6354.7	−6592.1	−6431.9	−6660.7	−6539.8	−6747.6
ρ=−0.8	DIC	12132.3	12517.4	12215.6	12672.1	12029.8	12461.9
	LPML	−6333.8	−6579.2	−6412.4	−6663.1	−6476.2	−6681.6

## Real Data Analysis

This study analyzed one dataset from the computer-based PISA 2018 (OECD, 2021) mathematics cognitive test with nine items in Albania, which was also used in the study by Shan and Wang (2020). According to the PISA 2018 (OECD, 2021) mathematics assessment framework, four attributes belonging to the mathematical content knowledge were assessed: change and relationship (α_1_), quantity (α_2_), space and shape (α_3_), and uncertainty and data (α_4_). Item responses were coded 0 (no credit), 1 (full credit), 6 (not reached), 7 (not applicable), 8 (invalid), and 9 (nonresponse). There were 798 examinees after removing examinees with codes 7 (not applicable) and 8 (invalid). In addition, 224 examinees with code 9 were also removed from this study because this study mainly focused on dropping-out missingness. Thus, the final sample was 574. The overall not-reached proportion was about 2%, and the not-reached proportions at the item level were from 0.7% to 3.3%. The item IDs and Q matrices are presented in Table 7.

**TABLE 7:** The Q matrix in the real data.

Attribute	CM033Q01	CM474Q01	CM155Q01	CM155Q04	CM411Q01	CM411Q02	CM803Q01	CM442Q02	CM034Q01
α_*1*_	0	0	1	1	0	0	0	0	0
α_*2*_	1	0	0	0	0	0	0	0	1
α_*3*_	0	1	0	0	1	0	0	1	0
α_*4*_	0	0	0	0	0	1	1	0	0

The DIC and LPML of the NMAR model in the real data were 5,760.28 and −3,040.03, respectively, and the DIC and LPML of the MAR model were 6,521.21 and −3,213.94, respectively. These two model fit indices indicated that the NMAR model fits the real data better than the MAR model. Thus, the NMAR model was adopted to fit this real dataset.

Tables 8, 9 show the estimated values and standard deviations of the item, person, and attribute parameters. Results show that the correlation coefficient of the person parameters is negative (i.e., −0.516), which indicates that the examinees with the higher abilities are less likely to drop out of the test. The estimated attribute slope parameters are positive, which implies that the knowledge attribute is better mastered with the increased ability $\theta_{i}^{h}$. The item mean parameter μ_β_ is estimated to be −1.749, which shows that the mean guessing probability is approximately 0.15. In addition, for the estimation of item parameters, only β_*j*_ for CM033Q01 is positive, while the β_*j*_ values for other items are negative, which implies that the guessing probability of item CM033Q01 is higher than 0.5 and the guessing probability of all other items is lower than 0.5. All δ_*j*_ are positive, which satisfies *g_j_* < 1−*s*_*j*_, as expected. Supplementary Figure 1 shows the proportions of attribute patterns for examinees with not-reached items, which illustrate that the most prevalent attribute pattern for examinees with not-reached items is (0000), which is unsurprising.

**TABLE 8:** Estimates and standard errors of the parameters for the real data.

Statistics	σ_*θ*^h^*θ*^d^_	$\sigma_{\theta^{d}}^{2}$	μ_β_	μ_δ_	$\sigma_{\beta }^{2}$	σ_βδ_	$\sigma_{\delta }^{2}$	λ_1_	λ_2_	λ_3_	λ_4_	γ_1_	γ_2_	γ_3_	γ_4_
Est.	−0.224	0.159	−1.749	2.380	3.058	−0.887	1.257	1.505	2.081	1.851	2.184	3.957	3.645	3.921	3.585
SD	0.149	0.040	0.379	0.292	2.108	1.241	0.979	0.399	0.427	0.443	0.382	0.441	0.432	0.446	0.482

*Est. is the estimated value, SD is the standard deviation.*

**TABLE 9:** Estimates and standard errors of the item parameters for the real data.

Parameter	Statistics	033Q01	474Q01	155Q01	155Q04	411Q01	411Q02	803Q01	442Q02	034Q01
β_*j*_	Est.	0.350	−0.251	−0.239	−1.213	−1.522	−1.296	−4.061	−4.325	−2.424
	SD	0.132	0.125	0.152	0.167	0.223	0.151	0.687	0.776	0.250
δ_*j*_	Est.	2.433	1.418	3.265	1.559	2.541	0.781	3.485	3.218	2.326
	SD	0.520	0.225	0.561	0.280	0.396	0.323	0.755	0.801	0.371

*Est. is the estimated value, SD is the standard deviation.*

## Conclusion

Not-reached items occurred frequently in cognitive diagnosis assessments. Missing data could help researchers understand examinees’ attributes, skills, or knowledge structures. Studies dealing with item nonresponses have used imputation approaches in cognitive diagnosis models, which may lead to biased parameter estimations. Shan and Wang (2020) introduced latent missing propensities of examinees for a cognitive diagnosis model that was governed by the potential category variables. However, their model did not distinguish the type of item nonresponses, which could result in inaccurate inferences regarding cognitive attributes and patterns.

In this study, a missing data model for not-reached items in cognitive diagnosis assessments was proposed. A DINA model was used as the response model, and a 1PLM was used as the missing indicator model. The two models were connected by two bivariate normal distributions for person parameters and item parameters. This new model was able to obtain more fine-grained attributes or knowledge structure as diagnostic feedback for examinees.

Simulation studies were conducted to evaluate the performance of the MCMC algorithm using the proposed model. The results showed that not-reached items provide useful information for further understanding the knowledge structure of examinees. Additionally, the HO-DINA model for the cognitive diagnosis assessments explained examinees’ cognitive processes, thus precise estimations of parameters were obtained from the proposed NMAR model. We compared the recovery of parameters under the two missing mechanisms, which revealed that the bias and RMSE of person parameters decreased significantly when using the proposed NMAR model when the missing proportion and the correlation of ability parameters were high. Moreover, considerable differences in the ACCRs and PCCRs between the NMAR and MAR models were found. With regard to model selection, the proposed NMAR model fitted the data better than the MAR model when the missing data mechanism was non-ignorable. The proposed NMAR model was successfully applied to the 2018 computer-based PISA mathematics data.

Several limitations of the study warrant mentioning, alongside future research avenues. First, this study only modeled not-reached items; however, examinees may skip the items in a cognitive test, which is another type of missing data that needs to be explored further. Second, missing data mechanisms in cognitive assessments may depend on individual factors, such as sex, culture, and race. In addition, different training and problem-solving strategies of examinees, and different school locations may also affect the pattern of nonresponses. Future studies can extend our model to account for the above-mentioned factors. Third, future studies could also incorporate the additional sources of process data, such as the response times, to explore the missing data mechanisms.

## Data Availability Statement

Publicly available datasets were analyzed in this study. This data can be found here: https://www.oecd.org/PISA/.

## Author Contributions

LL completed the writing of the article. JL provided the original thoughts. LL and JL provided key technical support. JZ, JL, and NS completed the article revisions. All authors contributed to the article and approved the submitted version.

## Conflict of Interest

The authors declare that the research was conducted in the absence of any commercial or financial relationships that could be construed as a potential conflict of interest.

## Publisher’s Note

All claims expressed in this article are solely those of the authors and do not necessarily represent those of their affiliated organizations, or those of the publisher, the editors and the reviewers. Any product that may be evaluated in this article, or claim that may be made by its manufacturer, is not guaranteed or endorsed by the publisher.
