# Supplementary material for: Modeling Not-Reached Items in Cognitive Diagnostic Assessments
Source: Front Psychol. 2022 Jun 13;13:889673. doi: 10.3389/fpsyg.2022.889673 (PMC9236559; doi:10.3389/fpsyg.2022.889673)
Supplement: Supplementary file 1 [file Data_Sheet_1.pdf]

Online Supplement

Modeling not-reached items in cognitive diagnostic assessments

Details of the MCMC algorithm of the proposed model

Let $\boldsymbol{\Omega} = \{\theta_i^h, \theta_i^d, \eta_0, \eta_1, \alpha_{ik}, \beta_j, \delta_j, \gamma_k, \lambda_k, \mu_\beta, \mu_\delta, \boldsymbol{\Sigma}_\mathbf{I}, \sigma_{\theta^h\theta^d}, \sigma_{\theta^d}^2\}$. The posterior distribution of the proposed model is expressed as follows:

$$p(\boldsymbol{\Omega}|\boldsymbol{Y}, \boldsymbol{D}) = \prod_{i=1}^{N}\prod_{j=1}^{J}\prod_{k=1}^{K} P(Y_{ij} = d)^{Y_{ij}=d} \times \{[1 - P(Y_{ij} = d)]P(Y_{ij} = 1)\}^{Y_{ij}=1}$$
$$\times \{[1 - P(Y_{ij} = d)]P(Y_{ij} = 0)\}^{Y_{ij}=0} \times p(\theta^h, \theta^d; \boldsymbol{\mu}_\mathbf{P}, \boldsymbol{\Sigma}_\mathbf{P})$$
$$p(\beta_j, \delta_j; \mu_\beta, \mu_\delta, \boldsymbol{\Sigma}_\mathbf{I}) p(\boldsymbol{\Sigma}_\mathbf{P}) p(\mu_\beta) p(\mu_\delta) p(\boldsymbol{\Sigma}_\mathbf{I}) p(\alpha_{ik}; \theta_i^h, \gamma_k, \lambda_k)$$
$$p(\lambda_k) p(\gamma_k) p(\eta_0) p(\eta_1).$$

Step 1: Renew β_j, δ_j for each j: Draw the candidate values $\begin{pmatrix}\beta_j^{(*)}\\ \delta_j^{(*)}\end{pmatrix} \sim N\left(\begin{pmatrix}\beta_j^{(r-1)}\\ \delta_j^{(r-1)}\end{pmatrix}, \Sigma_I^{(r-1)}\right)$.

Following Patz and Junker (1999), a M-H algorithm is employed, and the acceptance rate is $min(1, R_{\beta,\delta})$, and

$$R_{\beta,\delta} = \frac{\pi_{\beta,\delta}(\beta_j^*, \delta_j^*)\prod_{i=1}^{N} p(Y_{ij}|\beta_j^*, \delta_j^*, \alpha_{ik}^{(r-1)})}{\pi_{\beta,\delta}(\beta_j^{(r-1)}, \delta_j^{(r-1)})\prod_{i=1}^{N} p(Y_{ij}|\beta_j^{(r-1)}, \delta_j^{(r-1)}, \alpha_{ik}^{(r-1)})},$$

where $\pi_{\beta,\delta}$ is the normal density of the prior for parameter $\boldsymbol{\beta}, \boldsymbol{\delta}$.

Step 2: Renew μ_β: Draw the candidate values $\mu_\beta^{(*)} \sim N\left(\mu_\beta^{(r-1)}, c_{\mu_\beta}^2\right)$ with the acceptance rate $min\left(1, R_{\mu_\beta}\right)$, and

$$R_{\mu_\beta} = \frac{\pi_{\mu_\beta}(\mu_\beta^*)\prod_{j=1}^{J} P(\beta_j^{(r)}, \delta_j^{(r)}|\mu_\beta^*, \mu_\delta^{(r-1)}, \boldsymbol{\Sigma}_I^{(r-1)})}{\pi_{\mu_\beta}(\mu_\beta^{(r-1)})\prod_{j=1}^{J} P(\beta_j^{(r)}, \delta_j^{(r)}|\mu_\beta^{(r-1)}, \mu_\delta^{(r-1)}, \boldsymbol{\Sigma}_I^{(r-1)})},$$

where π_{μ_β} is the normal prior for parameter μ_β.

Step 3: Renew μ_δ: Draw the candidate values $\mu_\delta^{(*)} \sim N(\mu_\delta^{(r-1)}, c_{\mu_\delta}^2)$ with the acceptance rate $min(1, R_{\mu_\delta})$, and

$$R_{\mu_\delta} = \frac{\pi_{\mu_\delta}(\mu_\delta^*)\prod_{j=1}^{J} P(\beta_j^{(r)}, \delta_j^{(r)}|\mu_\beta^{(r)}, \mu_\delta^*, \boldsymbol{\Sigma}_I^{(r-1)})}{\pi_{\mu_\delta}(\mu_\delta^{(r-1)})\prod_{j=1}^{J} P(\beta_j^{(r)}, \delta_j^{(r)}|\mu_\beta^{(r)}, \mu_\delta^*, \boldsymbol{\Sigma}_I^{(r-1)})},$$

where π_{μ_δ} is the normal prior for parameter μ_δ.

Step 4: Renew $\boldsymbol{\Sigma}_\mathrm{I}$: $\boldsymbol{\Sigma}_\mathrm{I}$ are sampled from their posterior distribution, given $\Psi = (\beta^{(r)}, \delta^{(r)})'$. The inverse-Wishart prior is,

$$\boldsymbol{\Sigma}_\mathrm{I} \sim \text{Inverse-Wishart } (\boldsymbol{\Sigma}_{\mathrm{I}0}^{-1}, v_{I0}),$$

the posterior distribution is

$$\boldsymbol{\Sigma}_\mathrm{I}|\Psi \sim \text{Inverse-Wishart } (\boldsymbol{\Sigma}_{I*}^{-1}, v_{I*}),$$

where

$$\boldsymbol{\Sigma}_{I*} = \Sigma_{I0} + S_\Psi + \frac{k_{I0}J}{k_{I0} + J}(\bar{\Psi} - \mu_{I0})(\bar{\Psi} - \mu_{I0})'$$
$$v_{I*} = v_{I0} + J,$$

and S_Ψ is defined as

$$S_\Psi = \sum_{j=1}^{J} \left(\Psi_j - \bar{\Psi}\right)\left(\Psi_j - \bar{\Psi}\right)'.$$

Step 5: Renew λ_k for each k: Draw the candidate values $\lambda_k^{(*)} \sim U(\lambda_k^{(r-1)} - c_\lambda, \lambda_k^{(r-1)} + c_\lambda)$. The acceptance rate is $min(1, R_\lambda)$, and

$$R_{\boldsymbol{\lambda}} = \frac{\boldsymbol{\pi}_\lambda(\lambda_k^*) \prod_{i=1}^{N} p\left(\alpha_{ik} | \lambda_k^*, \gamma_k^{(r-1)}, \theta h_i^{(r-1)}\right)}{\boldsymbol{\pi}_\lambda\left(\lambda_k^{(r-1)}\right) \prod_{i=1}^{N} p\left(\alpha_{ik} | \lambda_k^{(r-1)}, \gamma_k^{(r-1)}, \theta h_i^{(r-1)}\right)},$$

where π_λ is the normal density of the prior for parameter $\boldsymbol{\lambda}$.

Step 6: Renew γ_k for each k: Draw the candidate values $\gamma_k^{(*)} \sim U(\gamma_k^{(r-1)} - c_\gamma, \gamma_k^{(r-1)} + c_\gamma)$. The acceptance rate is $min\left(1, R_\gamma\right)$, and

$$R_\gamma = \frac{\boldsymbol{\pi}_\gamma(\gamma_k^*) \prod_{i=1}^{N} p\left(\alpha_{ik} | \lambda_k^{(r)}, \gamma_k^*, \theta h_i^{(r-1)}\right)}{\boldsymbol{\pi}_\gamma\left(\gamma_k^{(r-1)}\right) \prod_{i=1}^{N} p\left(\alpha_{ik} | \lambda_k^{(r)}, \gamma_k^{(r-1)}, \theta h_i^{(r-1)}\right)},$$

where π_γ is the normal density of the prior for parameter $\boldsymbol{\gamma}$.

Step 7: Renew $\boldsymbol{\theta}_i = \begin{pmatrix} \theta_i^h \\ \theta_i^d \end{pmatrix}$ for each i: Draw the candidate values $\boldsymbol{\theta}_i^{(*)} \sim N\left(\begin{pmatrix} \theta_i^{h(r-1)} \\ \theta_i^{d(r-1)} \end{pmatrix}, \boldsymbol{\Sigma}_{\mathrm{P}}^{(\mathrm{r}-1)}\right)$ with the acceptance rate $min(1, R_\theta)$, and

$$R_\theta = \frac{\pi_\theta(\boldsymbol{\theta}_i^*) \prod_{j=1}^{J} \prod_{k=1}^{K} p\left(\alpha_{ik} | \lambda_k^{(r)}, \gamma_k^{(r)}, \boldsymbol{\theta}_i^{h*}\right) p\left(d_{ij} | \eta_0^{(r-1)}, \eta_1^{(r-1)}, \boldsymbol{\theta}_i^{d*}\right)}{\pi_\theta\left(\boldsymbol{\theta}_i^{(r-1)}\right) \prod_{j=1}^{J} \prod_{k=1}^{K} p\left(\alpha_{ik} | \lambda_k^{(r)}, \gamma_k^{(r)}, \boldsymbol{\theta}_i^{h(r-1)}\right) p\left(d_{ij} | \eta_0^{(r-1)}, \eta_1^{(r-1)}, \boldsymbol{\theta}_i^{d(r-1)}\right)},$$

where π_θ is the bivariate normal prior of parameter $\boldsymbol{\theta}$, and $\pi_\theta(\boldsymbol{\theta}_i^*) = p\left(\theta_i^{h*}, \theta_i^{d*} | \boldsymbol{\mu}_{\mathrm{P}}, \boldsymbol{\Sigma}_{\mathrm{P}}^{(r-1)}\right)$.

Step 8: Renew η_0: Draw the candidate values $\eta_0^{(*)} \sim N\left(\eta_0^{(r-1)}, c_{\eta_0}\right)$ with the acceptance rate $min\left(1, R_{\eta_0}\right)$, and

$$R_{\eta_0} = \frac{\pi_{\eta_0}(\eta_0^*) \prod_{i=1}^{N} \prod_{j=1}^{J} p\left(d_{ij} | \eta_0^*, \eta_1^{(r-1)}, \theta_i^{d(r)}\right)}{\pi_{\eta_0}\left(\eta_0^{(r-1)}\right) \prod_{i=1}^{N} \prod_{j=1}^{J} p\left(d_{ij} | \eta_0^{(r-1)}, \eta_1^{(r-1)}, \theta_i^{d(r)}\right)},$$

where π_{η_0} is the normal prior for parameter η_0.

Step 9: Renew η_1: Draw the candidate values $\eta_1^{(*)} \sim N\left(\eta_1^{(r-1)}, c_{\eta_1}\right)$ with the acceptance rate $min\left(1, R_{\eta_1}\right)$, and

$$R_{\eta_1} = \frac{\pi_{\eta_1}(\eta_1^*) \prod_{i=1}^{N} \prod_{j=1}^{J} p\left(d_{ij} | \eta_0^{(r)}, \eta_1^*, \theta_i^{d(r)}\right)}{\pi_{\eta_1}\left(\eta_1^{(r-1)}\right) \prod_{i=1}^{N} \prod_{j=1}^{J} p\left(d_{ij} | \eta_0^{(r)}, \eta_1^{(r-1)}, \theta_i^{d(r)}\right)},$$

where π_{η_1} is the normal prior for parameter η_1.

Step 10: Renew $\boldsymbol{\alpha}_i$: Draw the candidate values $\alpha_{ik}^{(*)} \sim Bernoulli(1, 0.5)$ $(k = 1, \cdots, K)$ with the acceptance rate $min(1, R_\alpha)$, and

$$R_\alpha = \frac{\pi_\alpha(\boldsymbol{\alpha}_i^*) \prod_{j=1}^{J} p\left(Y_{ij} | \beta_j^{(r)}, \delta_j^{(r)}, \boldsymbol{\alpha}_i^*\right)}{\pi_\alpha\left(\boldsymbol{\alpha}_i^{(r-1)}\right) \prod_{j=1}^{J} p\left(Y_{ij} | \beta_j^{(r)}, \delta_j^{(r)}, \boldsymbol{\alpha}_i^{(r-1)}\right)},$$

where π_α is the prior for parameter $\boldsymbol{\alpha}$, $\pi_\alpha(\boldsymbol{\alpha}_i^*) = \prod_{k=1}^{K} p(\boldsymbol{\alpha}_i^*; \lambda_k^{(r)}, \gamma_k^{(r)}, \theta_i^{h(r)})$.

Step 11: Renew the covariance $\sigma_{\theta^h \theta^d}$. The new value $\sigma_{\theta^h \theta^d}^*$ is sampled from a truncated normal distribution $N\left(\sigma_{\theta^h \theta^d}^{(r-1)}, c_{01}^2\right) I\left(-p_{01} = -\sqrt{\sigma_{\theta^d}^{2(r-1)}} < \sigma_{\theta^h \theta^d}^* < \sqrt{\sigma_{\theta^d}^{2(r-1)}} = p_{01}\right)$ with the acceptance rate $min\left(1, R_{\sigma_{\theta^h \theta^d}}\right)$, and

$$R_{\sigma_{\theta^h\theta^d}} = \frac{\prod_{i=1}^{N} p\left(\theta_i^{d(r)} \middle| \theta_i^{h(r)}, \sigma_{\theta^d}^{2(r-1)}, \sigma_{\theta^h\theta^d}^{*}\right) p\left(\sigma_{\theta^h\theta^d}^{*}\right)\left(\Phi\left(\frac{p_{01}-\sigma_{\theta^h\theta^d}^{(r-1)}}{c_{01}}\right)-\Phi\left(\frac{-p_{01}-\sigma_{\theta^h\theta^d}^{(r-1)}}{c_{01}}\right)\right)}{\prod_{i=1}^{N} p\left(\theta_i^{d(r)} \middle| \theta_i^{h(r)}, \sigma_{\theta^d}^{2(r-1)}, \sigma_{\theta^h\theta^d}^{(r-1)}\right) p\left(\sigma_{\theta^h\theta^d}^{(r-1)}\right)\left(\Phi\left(\frac{p_{01}-\sigma_{\theta^h\theta^d}^{*}}{c_{01}}\right)-\Phi\left(\frac{-p_{01}-\sigma_{\theta^h\theta^d}^{*}}{c_{01}}\right)\right)},$$

where $p\left(\theta_i^d \middle| \theta_i^h\right)$ is the conditional density function, and $p\left(\sigma_{\theta^h\theta^d}\right)$ is a uniform prior density function.

Step 12: Renew $\sigma_{\theta^d}^2$. Draw the candidate values $\sigma_{\theta^d}^{2*}$ from a truncated normal distribution $N\left(\sigma_{\theta^d}^{2(r-1)}, c_{02}^2\right) I\left(\sigma_{\theta^d}^{2*} > \left(\sigma_{\theta^h\theta^d}^{*}\right)^2 = p_0\right)$ with the acceptance rate $min\left(1, R_{\sigma_{\theta^d}^2}\right)$, and

$$R_{\sigma_{\theta^d}^2} = \frac{\prod_{i=1}^{N} p\left(\theta_i^{d(r)} \middle| \theta_i^{h(r)}, \sigma_{\theta^d}^{2*}, \sigma_{\theta^h\theta^d}^{(r)}\right) p\left(\sigma_{\theta^d}^{2*} | v_0, c_0\right)\left(1-\Phi\left(\frac{p_0-\sigma_{\theta^d}^{2(r-1)}}{c_{02}}\right)\right)}{\prod_{i=1}^{N} p\left(\theta_i^{d(r)} \middle| \theta_i^{h(r)}, \sigma_{\theta^d}^{2(r-1)}, \sigma_{\theta^h\theta^d}^{(r)}\right) p\left(\sigma_{\theta^d}^{2(r-1)} | v_0, c_0\right)\left(1-\Phi\left(\frac{p_0-\sigma_{\theta^d}^{2*}}{c_{02}}\right)\right)},$$

where $p\left(\sigma_{\theta^d}^2 | v_0, c_0\right)$ is the density function of the inverse Gamma distribution with shape parameter v_0 and the scale parameter c_0.

Table S1. ACCRs and PCCRs of three models under different correlations between $\boldsymbol{\theta}^h$ and $\boldsymbol{\theta}^d$ and different dropping-out proportions in simulation study III.

		Low dropping-out proportion			Medium dropping-out proportion			High dropping-out proportion		
ρ		NMAR	MAR	HO-DINA	NMAR	MAR	HO-DINA	NMAR	MAR	HO-DINA
0	ACCR	0.962	0.961	0.961	0.959	0.960	0959	0.952	0.952	0.952
		0.982	0.982	0.982	0.984	0.984	0.984	0975	0.975	0.975
		0.983	0.983	0.983	0.981	0.981	0.981	0.971	0.971	0.971
		0.986	0.986	0.986	0.985	0.985	0.985	0.976	0.976	0.977
		0.985	0.985	0.985	0.983	0.982	0.983	0967	0.967	0.967
	PCCR	0.909	0.909	0.909	0.904	0.904	0.904	0.865	0.866	0.866
−0.5	ACCR	0.963	0.941	0.942	0.961	0.937	0.937	0.952	0.907	0.905
		0.982	0.979	0.979	0.981	0.977	0.977	0.975	0.969	0.969
		0.983	0.980	0.980	0.983	0.977	0.976	0.972	0.963	0.962
		0.986	0.984	0.984	0.984	0.981	0.981	0.977	0.970	0.970
		0.984	0.979	0.979	0.984	0.980	0.981	0.971	0.964	0.963
	PCCR	0.910	0.883	0.883	0.905	0.871	0.871	0.871	0.814	0.812
−0.8	ACCR	0.964	0.941	0.941	0.962	0.932	0.930	0.955	0.902	0.902
		0.983	0.979	0.979	0.981	0.977	0.977	0.974	0.969	0.970
		0.983	0.978	0.978	0.983	0.977	0.977	0.970	0.960	0.961
		0.987	0.984	0.985	0.985	0.982	0.982	0.977	0.971	0.972
		0.987	0.984	0.984	0.983	0.979	0.979	0.969	0.962	0.962
	PCCR	0.915	0.883	0.883	0.906	0.869	0.868	0.873	0.812	0.814

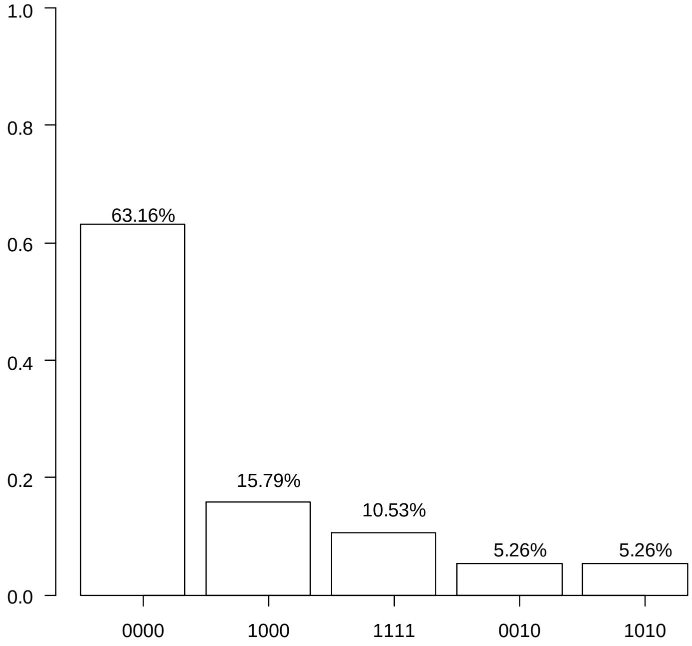

Figure S1. Proportions of attribute patterns of examinees with not-reached items in the real data.
